# The influence of environmental factors on the generalisability of public health research evidence: physical activity as a worked example

**DOI:** 10.1186/1479-5868-8-128

**Published:** 2011-11-16

**Authors:** Paul Watts, Gemma Phillips, Mark Petticrew, Angela Harden, Adrian Renton

**Affiliations:** 1 Institute for Health and Human Development, University of East London, Water Lane, London, E15 4LZ, UK; 2 Department of Social and Environmental Health Research, London School of Hygiene and Tropical Medicine, 15-17 Tavistock Place, London, United Kingdom, WC1E 9SH, UK

**Keywords:** generalisability, physical activity, environment, applicability, transferability, external validity, settings, public health

## Abstract

**Background:**

It is rare that decisions about investing in public health interventions in a city, town or other location can be informed by research generated in that specific place. It is therefore necessary to base decisions on evidence generated elsewhere and to make inferences about the extent to which this evidence is generalisable to the place of interest. In this paper we discuss the issues involved in making such inferences, using physical activity as an example. We discuss the ways in which elements of the structural, physical, social and/or cultural environment (environmental factors [EFs]) can shape physical activity (PA) and also how EFs may influence the effectiveness of interventions that aim to promote PA. We then highlight the ways in which EFs may impact on the generalisability of different types of evidence.

**Discussion:**

We present a framework for thinking about the influence of EFs when assessing the generalisability of evidence from the location in which the evidence was generated (place A) to the location to which the evidence is to be applied (place B). The framework relates to similarities and differences between place A and place B with respect to: a) the distributions of EFs; b) the causal pathways through which EFs or interventions are thought to exert their effect on PA and c) the ways in which EFs interact with each other. We suggest, using examples, how this scheme can be used by public health professionals who are designing, executing, reporting and synthesising research on PA; or designing/implementing interventions.

**Summary:**

Our analysis and scheme, although developed for physical activity, may potentially be adapted and applied to other evidence and interventions which are likely to be sensitive to influence by elements of the structural, physical, social and/or cultural environment such as the epidemiology of obesity and healthy weight promotion.

## Introduction

It is widely advocated that decisions about investment in public health interventions should be based on the best available research evidence. This evidence will preferably include a high quality randomised controlled trial (RCT) of the intervention under consideration, carried out in the area targeted for public health action. If an RCT has not been conducted, information from observational studies conducted in that place may also provide useful information. However, this location-specific evidence is seldom available and therefore decisions must be based on evidence from studies that were conducted elsewhere [1]. Under these circumstances, it is necessary to make inferences about the generalisability of the research evidence from the location in which it was generated to the location of interest.

Regular physical activity is effective in reducing the risk of premature death and in preventing the development of many chronic diseases [2]. It is currently recommended that adults take some moderate or vigorous physical activity every day and a minimum of 150 minutes per week to accrue such health benefits [3]. However, less than half of adults in the UK meet these minimum recommended levels [4]. Traditionally, interventions for increasing physical activity levels have focused on individual behaviours, using "information, education and communication" approaches [5]. However, there is increasing recognition that structural (e.g. government policies), physical (e.g. the built environment), social and cultural factors may also influence individual physical activity level [6]. We will refer to the range of physical, social, cultural and political characteristics of a location as "environmental factors". There is now great interest in interventions that aim to directly modify environmental factors to provide supports for, or remove barriers to, groups of individuals increasing their physical activity levels. These interventions range from physical modifications of the urban environment to changes in policies on the pricing and provision of public transport or recreational facilities [7]. This broadening of the focus of public health action to include environmental and structural interventions is mirrored for other health behaviours and outcomes [8].

However, if environmental factors are important determinants of health behaviours, they may also moderate the effects of any interventions that are implemented. This is true whether the interventions seek to modify health behaviours through manipulation of environmental factors or through information, education and communication approaches directed at individuals. Given the very large variation in environmental factors between countries, regions or municipalities, an intervention that works well in one location could have a different effect, or no effect at all, when transferred to another location [9]. It is therefore essential to carefully consider the differences in environmental factors when deciding whether it is appropriate to generalise research evidence on the determinants of health behaviours, such as physical activity, and associated interventions to a new location.

In this paper we briefly review the discussion of the generalisability of public health research evidence in the peer-reviewed literature and describe how environmental factors fit into the broader issue of generalisability. We then describe the types of research evidence on physical activity determinants and interventions that particularly require consideration of environmental factors when making decisions on generalisability and the current state of guidance on how to make these decisions. Building on this brief review, we develop a causal model to describe how inter-setting variation in environmental factors, and the causal pathways through which environmental factors affect physical activity, could critically influence the generalisability of research evidence on physical activity determinants and interventions. Finally, we present a framework for considering environmental factors in the assessment of research evidence generalisability, based on the causal model, and outline the implications of this framework for: the interpretation of existing evidence; the design, execution, reporting and synthesis of research in this field; and the design and implementation of interventions to promote physical activity.

## Background

### Generalisability in the peer-reviewed literature

There is a considerable body of literature addressing whether findings of a particular research study might be reproducible in other populations or settings, but the language and terminology used by epidemiologists, behaviour change experts, policy makers and social advocates varies greatly, both within and across disciplines. Wang and colleagues [1] reviewed this literature, finding that the terms 'applicability', 'generalisability' and 'transferability' are often used interchangeably, with no consensus over their meaning. They suggest that in most cases, these terms are used to describe the extent to which findings are generally useful beyond the original context in which they were generated. Wang and colleagues suggest a new definition of these terms where 'applicability' indicates whether the intervention could practically be implemented in the new setting, therefore focussing on the *process *of implementation, whilst 'transferability' indicates whether the intervention can be expected to have the same effect in the new setting, therefore focussing on the *outcome *of the intervention. In this taxonomy generalisability is a synonym for transferability.

**Figure 1:**
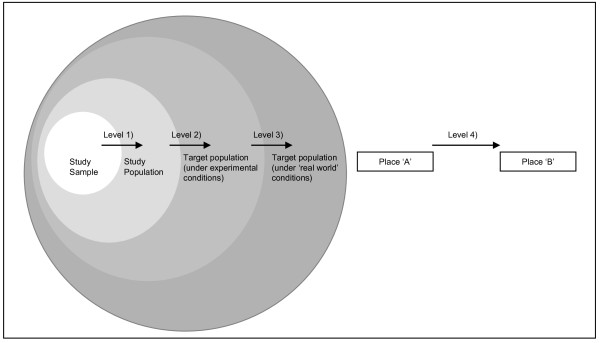
**Levels of inference from research studies**.

There is more consistency in the use of the term 'external validity', which is used to denote the extent to which inferences about relationships and effects can be made beyond the range of settings, populations or time periods sampled in the original research study. The scheme presented by Shadish et al [10] can be extended to describe four levels at which inferences about the extrapolatability of evidence are commonly made in research studies (illustrated in Figure 1):

- Level 1: from the study sample to the study population;

- Level 2: from the study population to the target population under experimental conditions;

- Level 3: from the target population under experimental conditions to the target population in 'real world' conditions;

- Level 4: from one location or environment to another.

The problems associated with making extrapolations at levels 1, 2 and 3 have been discussed extensively in the literature regarding RCTs for individual-level medical interventions [11]. However, there has been far less discussion of level 4, the extrapolatability of evidence generated in one place or environment to another [12]. It is at this level, the transfer of interventions or evidence from one setting to another, that consideration of environmental factors is essential. In this paper we will address this inter-setting extrapolation of research evidence, which corresponds to external validity in relation to broader and different populations described by Shadish et al [10] and to 'transferability' described by Wang et al [1]. We reserve the term 'generalisability' to refer to confidence in making inter-setting/level 4 inferences from research findings.

### Typology of research evidence on physical activity determinants and interventions

#### Evidence typology

There are three main types of evidence relevant to the promotion of physical activity whose generalisability is likely, *a priori*, to be significantly affected by inter-setting variation in elements of the structural, physical, social and cultural environment.

Type 1 Evidence: Evidence on the influence of environmental factors on physical activity (observational studies).

Type 2 Evidence: Evidence of the effectiveness of interventions that seek to increase physical activity levels of individuals by providing information, education and/or communication approaches (IEC-based interventions).

Type 3 Evidence: Evidence of the effectiveness of interventions that seek to increase physical activity levels of individuals by modifying one or more elements of the structural, physical, social and cultural environment and which may incorporate information, education and/or communication components in a complex intervention (environment-based interventions).

#### Type 1 evidence: The influence of environmental factors on physical activity (observational studies)

Many public health bodies and officials have recognised the importance of environmental factors in influencing the physical activity levels of populations including the Chief Medical Officer for England and Wales [13], the Office for Science Foresight Report on Obesity [14], the National Institute for Health and Clinical Excellence (NICE) guidance [7] and the Strategic Review of Health Inequalities in England [15]. Over 400 studies, within disciplines that include transport studies, urban-design, public health and epidemiology, have described associations between different combinations of environmental factors and physical activity levels. Several comprehensive reviews of this evidence base have summarised the key relationships [6,16-18]. A summary of environmental factors consistently reported to be associated with physical activity is presented in Table 1 where environmental factors are categorised according to whether they pertain to structural, physical, social or cultural aspects of the environment. References to the reviews in which each association is reported have been provided. The majority of research to date has focused on physical environmental factors, therefore we have also included in Table 1 social and cultural environmental factors that have been under-researched [16] or excluded from reviews of the literature as published studies have used qualitative methods [19-22].

**Table 1:** Environmental factors associated with physical activity

Structural Environment	Physical Environment	Social Environment	Cultural Environment
• Traffic [6,18]	• Incivilities (graffiti, litter etc) [18]	• Traffic safety [18]	• PA levels of others [18]
• Public transport accessibility [18]	• Hilliness [6,18]	• Perceived Safety [6,18]	• Racial discrimination
• Affordable, accessible, good quality recreation facilities [6,16-18]	• Perceived aesthetics [6]	• Social cohesion [17,18]	• Acceptable clothing
• Amenities that facilitate walking [6,18]	• Weather/temperature [17,18]	• Social support [17-19]	• Attitudes towards physical activity
• Connectivity, road and path networks, cycle lanes [6,18]	• Land use mix [6]	• Anti-social behaviour	• Ethnic/Cultural preferences for certain activities
• Presence of sidewalks, controlled crossings [6,18]	• Air/noise pollution [18]	• Neighbourhood deprivation [17]	• Cultural activities (e.g. dancing)
• Residential density/Population density [6]		• PA levels of others [18]	• Religious practices (e.g. holidays, activities)
• Urbanity/Age of area [6,17,19]		• Racial discrimination	• Culturally specific understandings of appropriate PA and its benefits
• Access to community/health facilities, services, organisations [6,16]			• Racial discrimination

#### Type 2 and 3 evidence: Interventions to promote physical activity

A wide range of interventions to promote physical activity has been described [16]. These interventions aim to directly modify individual behavioural choices, or to modify the environmental factors that may condition these choices.

The majority of research into physical activity promotion has focused on policies and interventions that seek to increase physical activity levels of individuals or populations directly through information, education and communication approaches. Systematic reviews have demonstrated short-term effectiveness for some classes of information, education and communication interventions [23,24] and a range of these is recommended by NICE in the UK [25]. A small number of interventions which aim to increase the physical activity levels of populations through modifications to environmental factors have been shown to be effective [26,27]. NICE has recently conducted a review of such interventions and has endorsed the use of a range of environment-based interventions which have been shown to be effective [7].

### Existing guidance for considering the inter-setting generalisability of evidence on physical activity determinants and interventions

#### Type 1 evidence: The influence of environmental factors on physical activity (observational studies)

In 2007 NICE conducted a synthesis of systematic reviews of observational studies investigating associations between environmental factors and physical activity [6] which highlighted the paucity of studies conducted in the UK. However, the authors did not discuss the generalisability of evidence from studies outside the UK to the UK context. It is likely that the authors' ability to comment on generalisability was limited by the absence of any in-depth discussion of generalisability in the systematic reviews they were synthesising and the primary studies contained in these reviews.

#### Type 2 evidence: Interventions to promote physical activity through information, education and communication

NICE has produced several sets of guidance informed by evaluations of the effectiveness of interventions employing information, education and communication approaches to increasing physical activity [25,28], which included assessment of the utility of reviewed evidence for public health decision-making in the UK. In this guidance, the term 'applicability' is used most often, but is used to indicate both the extent to which inferences can be made about both the generalisability of evidence of effectiveness generated outside the UK to the UK (analogous to 'transferability' as described by Wang et al [1] and our definition of generalisability) and the extent to which practical issues may act as barriers to implementing the intervention in the UK (analogous to 'applicability' as described by Wang et al [1]). It is often unclear which of these meanings is intended and there is no description of the methods used to make judgements about these issues.

NICE have since published an updated description of the methods used in developing their guidance [29], in which they clearly distinguish between the successful transfer of intervention processes and the replication of intervention outcomes. In relation to applying international evidence in the UK, they suggest that one should consider whether there are any 'demographic or geographic' factors that might influence generalisability. However, there is no guidance on assessing how this influence might be exerted or predicting in what ways generalisability might be affected. In relation to practical issues in implementation, NICE suggests that a range of structural, social, cultural and demographic factors (including some that we have presented in Table 1) should be considered. Examination of the 'geographical context' in which the intervention was originally implemented is recommended, but 'rural/urban' is the only operationalisation provided. The guidance also follows Bonell et al [12] who suggest that assessing the practical 'applicability' of an intervention requires consideration of 'feasibility', 'acceptability' and 'capacity' in both the place the intervention was originally delivered and alternative places the intervention might be implemented.

#### Type 3 evidence: Interventions to promote physical activity through modification of environmental factors

In 2008 NICE produced a review of evidence focussing on interventions in which environmental factors are modified in order to influence physical activity levels [7]. The resulting guidance again highlighted the predominance in the literature of research conducted outside the UK, and concluded that the range of participants included might not therefore reflect the sociodemographic diversity of some areas in the UK, and also that the distribution of environmental factors in the intervention trial sites (commonly suburban areas in the US and Australia) might not be comparable to the distribution of environmental factors found in the UK. Whilst the guidance does discuss generalisability, the methods used to assess it are not clearly described. As NICE guidance on environmental interventions [7] was produced before publication of the methodological guidance discussed above (under 'Type 2 evidence'), we sought clarification from the authors. We were informed by the lead analyst [30] that both the review team and the programme development group considered and discussed issues including the country in which the research was conducted, the date, cultural differences and urban/rural variation in population attribute and the environment. Judgements were made based on these discussions and the expertise within the programme development group.

As described in our evidence typology, complex environment-based interventions may incorporate information, education and communication components. The Medical Research Council guidance on developing and evaluating complex interventions [9], does not provide guidance on assessing generalisability, but does acknowledge that 'context is crucial' because interventions that are effective in one place may have a different or no effect in another. Rychetnik et al [31] produced a set of criteria for evaluating evidence on complex public health interventions in which they also highlight the importance of 'context', which they define as *"the social, political and/or organisational setting in which an intervention was evaluated, or in which it is to be implemented" *(p 119). These authors suggest that in assessing generalisability, information is needed on: the context of the intervention; the design and components of the intervention; and any potential interactions between the intervention and its context. They provide useful examples which illustrate why this information is important in assessing generalisability. This work was built upon by Wang et al [1] who further suggest that information is required regarding the prevalence of the health problem in question, the political and social environment, organisational structure and resources in the original research setting and the setting to which the evidence is to be generalised. Furthermore, Wang et al suggest that this information may be collected using Delphi studies involving professionals with diverse expertise, using consultations with people who have an in depth knowledge of the setting to which the evidence is to be generalised or using information gathered from systematic searches of the available literature.

Green and Glasgow [32], have provided a very useful summary of literature that has sought to address issues relating to the generalisability of public health research and have highlighted the importance of frameworks that consider generalisability between settings as well as between individuals. Following this summary, Green and Glasgow present criteria for the assessment of the generalisability of public health research. This includes the assessment of similarities and differences between the populations in which research has been conducted and the populations to which the evidence or intervention is to be applied (participation rates, target audiences, representativeness of participants, drop-out rates), similarities and differences in the implementation of the program (consistency of implementation, staff expertise, program adaptation, mechanisms through which the program exerted it's effect, sustainability and long term effects) and the quality of information provided in research reports regarding methods and outcomes (comparability of outcomes, adverse consequences, moderator effects, sensitivity and cost analysis).

In addition to these criteria, Green and Glasgow highlight the importance of comparing similarities and differences between settings; suggesting that the size, level of urbanity and availability of resources need to be considered when assessing generalisability. However, the authors also recommend development of these criteria and the formulisation of new criteria for making judgements about generalisability. In this paper, we aim to draw attention to factors influencing inter-setting generalisability that have not been discussed in any detail in the literature described above. This will include the comprehensive consideration of the ways in which the range of environmental factors presented in Table 1 may influence generalisability.

**Figure 2:**
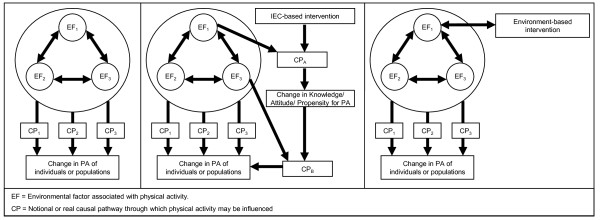
**Model to show environmental factors and causal pathways for different types of evidence**.

Despite repeated exhortations to consider issues of inter-setting generalisability in relation to observational studies and intervention studies in the field of physical activity, the above review highlights that there is little guidance in the literature to suggest how this might actually be achieved. In the following section we develop a causal model (Figure 2) that describes how environmental factors might interact to influence the findings of studies which generate evidence about physical activity determinants and interventions and then develop a framework for considering the influence of environmental factors in the assessment of the generalisability of this research evidence.

## Discussion

### Development of a framework for assessing the inter-setting generalisability of evidence on physical activity determinants and interventions

#### Causal model for the interaction between environmental factors and research findings

##### Type 1 Evidence: Observational Studies

The left panel of Figure 2 depicts the relationships between environmental factors and physical activity levels that have been identified in observational research studies. Environmental factors may exert direct mutual influence on each other as well as interacting in one or more causal pathways which directly influence physical activity levels. For example, observed associations between residential density (EF_1_) and physical activity [33] are likely to be dependent on public transport accessibility levels (EF_2_) and the thresholds for residential density that are used in many countries to trigger the introduction of bus services (EF_3_)[34]. These three environmental factors may vary greatly between settings, but the ways in which they influence each other may also vary. Residential density has an important influence on walkability which may be expected to directly influence physical activity levels [35]. However, bus services may influence physical activity by modifying perceptions of walkability (will I still consider 20 minutes to my friend's house walkable if I can do it by bus in 5 minutes?) or by another mechanism such as enabling easy access to facilities previously out of range[36]. Thus, observed associations between residential density and physical activity [7] from the USA (where 18 dwellings per hectare is enough to justify a local bus service), may not be consistent in a setting where this threshold is different (such as the UK where a density of 25 dwellings per hectare is considered too sparse to be able to maintain a bus service) [34].

##### Type 2 Evidence: Interventions to promote physical activity through information, education and communication

The centre panel shows the situation for studies of interventions which seek to modify behaviour by providing information, education or communication. Again, environmental factors may exert direct mutual influence on each other and interact in causal pathways to influence physical activity levels. For example, high levels of crime and vandalism (EF_1_) will have a negative effect on the perceived aesthetic quality of the environment (EF_2_) and the perceived safety of the environment (EF_3_). In addition environmental factors may interact with the causal pathways through which information, education and communication activities either achieve changes in knowledge about, attitudes to or propensities for physical activity (CP_A_), or the pathways through which changes in these are translated into changes in physical activity (CP_B_). An example of how these pathways may influence the effect of an interviention is provided by Michael and Carlson [37] who measured the moderating effect of environmental factors on information-based walking interventions in Oregon, US, finding that perceived neighbourhood problems (gangs, graffiti, violent crime, vandalism, burglary, abandoned or boarded up buildings, or alcohol or drug use) appeared to suppress the effect of the intervention, while measures of social cohesion and neighbourhood walkability (physical-environment characteristics) were not significant moderators of the intervention effect. Therefore, an evaluation of the same intervention, implemented in a setting with different configurations of these environmental factors may produce a different outcome.

##### Type 3 Evidence: Interventions to promote physical activity through modification of environmental factors

The right panel depicts studies of interventions acting on environmental factors. In this case, the direct mutual influence of environmental factors on each other may constrain or enhance the ability of the intervention to secure the desired changes. For example, the effect of an intervention designed to create and maintain outdoor environments containing serviceable exercise equipment (EF_1_) may be constrained by high levels of crime and vandalism in an area (EF_2_) [38]. Further, other environmental factors (which are not the primary targets for the intervention) such as street connectivity (EF_3_) [33] may influence the both the ability of individuals to access the exercise equipment provided and the levels of crime and vandalism in the area. Therefore, the outcome of an evaluation of the same intervention in a different setting may be different.

The studies cited above provide information about the evidence base for the proposed causal pathways. However, further research is required to generate evidence to support all causal pathways proposed in our model. This includes investigation of: (1) the under-researched environmental factors listed in Table 1 (predominantly social and cultural factors); (2) the ways in which environmental factors interact to influence physical activity; (3) the ways in which environmental factors influence each other independently of physical activity.

### Framework for considering environmental factors in assessment of the generalisability of existing research evidence

Following from the causal model of environmental influences on physical activity determinants and interventions, we suggest that three principal considerations are necessary when generalising evidence generated in one location (place A) to another location (place B), independently of whether this evidence relates to observational studies, studies of the effect interventions. The three domains of the framework are;

*1. The configuration of environmental factors in places A and B and the differences between these*.

*2. The actual or notional causal pathways through which environmental factors exert their effect on PA in place A and in place B and the differences between these*.

*3. The ways in which different environmental factors influence each other in place A and place B and the differences between these*.

In what follows we now present some practical ways in which these three domains can be systematically considered.

*1. The configuration of environmental factors in places A and B and the differences between these*.

In order to assess the configuration of environmental factors in places A and B and the differences between these, it is first necessary to decide which of the environmental factors listed in Table 1 are likely to influence physical activity and/or the processes of the intervention (if applicable) in places A and/or B. Secondly, appropriate sources of information about these environmental factors should be identified. In some cases, information about environmental factors in place A may be available from the published reports of the evidence to be generalised. If not, in the first instance we suggest contacting the authors of these reports as they are likely to be best placed to provide (or suggest sources of) this information. Alternatively, information about environmental factors in place A can be sought by accessing routinely available data sets where available.

Cummins et al [39] have provided an overview of the types of appropriate routine data that may be available and the ways in which it may be accessed and operationalised. Here, we will give an overview of how some of the factors listed in Table 1 may be evidenced from routine data. The following examples are from England and Wales, but similar data are available in many other countries, and the methods we describe can be applied in most countries. In England and Wales, routinely available data sets include those provided by the Office for National Statistics [40], and the large range of publically available data from diverse sources accessible through the single government data repository website [41]. These sources provide localised (census lower/middle super output areas) and local authority-level data on a wide range of socioeconomic factors as well as environmental factors including traffic, public transport accessibility, traffic safety, air/noise pollution, hilliness. Information regarding road and path networks and hilliness are available from Great Britain's national mapping agency, Ordnance Survey [42] and information about cycle lanes from UK charity Sustrans [43]. Measures such as street connectivity are not as readily available, but simple indicators of connectivity can be derived by counting the number of streets and street intersections and using one of several methods for calculating a connectivity index [44]. Resources for physical activity can be identified using Sport England's 'Active Living Database' [45]. Similarly, relevant, locally-specific health statistics from these sources such as obesity rates, healthy eating measures and rates of physical activity may help assessment of the extent to which the needs of people in place A and place B differ [31].

Beyond routine datasets, there is a range of robust quantitative assessment tools available that can be used to assess amenities that facilitate walking including the presence and quality of sidewalks. A selection (though mostly designed in relation to US neighbourhoods) can be found on the Active Living Research website [46]. Additionally, consultation with 'local experts' is likely to be valuable in order to gather information about the social and cultural factors listed in Table 1 where routine data sets are not available.

Once information about the configuration of environmental factors in place A and place B has been collated, a first step is to judge which environmental factors show potentially significant inter-setting differences in the light of the wider evidence base. Next, it is necessary to assess whether these differences are likely to influence generalisability. This can be approached by considering how each environmental factor showing potentially significant inter-setting differences might influence physical activity directly, or the processes of the intervention (if applicable). Then each environmental factor can be rated according to the extent of its likely influence on generalisability, to inform an overall judgement. In Table 2 we present an example of how this might be achieved. This is for illustration and is not proposed as a rigid framework. Each case is likely to be different and methods will need to be adapted. For example, it may be appropriate to differentially weight the ratings of some environmental factors if some are considered to be particularly important when making the overall judgement.

**Table 2:** An illustrative example of how differences between configurations of environmental factors may be assessed.

**Evidence to be generalised**.	**Evidence for increased levels of PA following the introduction of an additional light rail stop from Brown & Werner **[51]	
**EFs that are known to be associated with PA and/or may influence the processes of the intervention**.	Street Connectivity	Population Density

**Information about 'place A' (neighbourhood in Salt Lake City) available from published report**.	*"The residential areas had gridded street patterns" *(High street connectivity)	None Available

**Other information about this EF in 'place A'**	None required	Location of place A could be identified by contacting the authors and population density identified by searching census data. Population density = 6.67 persons per hectare.

**Information about 'place B' (A neighbourhood in London)**	Street connectivity could be assessed using methods described by Ewing [32] found place B to have med/low connectivity.	Population density identified by searching census data. Population density = 47.57 persons per hectare

**Information needed to make judgements about the extent to which differences in this EF between places A and B may influence the generalisablity of this evidence**	Research [32] has shown that higher levels of street connectivity are associated with higher levels of PA. Furthermore, low levels of street connectivity reduce access to destinations, such as the rail stop that is the focus of the intervention.	Research [52] has shown that modest differences in population density (smaller than that we have identified between places A and B) are associated with differences in PA levels.

**On a scale of '1' (very likely to negatively influence PA or intervention processes) to '5' (very unlikely) rate the extent to which differences between this EF in place A and place B are likely to influence PA levels or the processes of the intervention (if applicable). Indicate what type of influence this is likely to be (e.g. positively/negatively influence PA levels)**.	The lower levels of street connectivity in place B are likely to negatively influence PA and specifically to influence accessibility to a rail stop. For these reasons, this EF is rated '2' - likely to influence PA and/or the intervention processes and therefore likely to be a barrier to generalising this evidence to place B	The higher population density in place B is likely to positively influence PA. For this reason, this EF is rated '5' - very unlikely to negatively influence PA levels and therefore unlikely to be a barrier to generalising this evidence to place B.

*2. The actual or notional causal pathways through which environmental factors exert their effect on PA in place A and in place B and the differences between these*.

Our current understanding of the causal pathways through which environmental factors exert their effect on physical activity is largely based on commonsense narratives rather than evidence. Developing and evidencing models that conceptualise these causal pathways has recently been identified as a priority by a working group of leading researchers [47]. They suggest that the lack of models may be a key a barrier to moving forwards to produce strong evidence of associations between environmental factors and physical activity, and of the effectiveness of environment-based interventions to increase physical activity levels. We further propose that this deficit is also a key challenge in assessing the inter-setting generalisability of such evidence.

In the diagrams presented in Figure 2 and the examples used to illustrate these, we have suggested ways in which environmental factors may interact with each other and/or with environment-based and information, education and communication-based interventions to exert their influence on physical activity. In order to make inferences about the generalisability of the three types of evidence we describe, it is necessary to make *a priori *judgements about the causal pathways through which environmental factors exert their effect on physical activity in places A and B. To do this we can start by considering whether any causal pathways or models which are described or hypothesised to explain the evidence reported from place A are likely also to be applicable in place B. As process and qualitative evaluations are increasingly encouraged [9] alongside quantitative descriptive and experimental studies, such pathways and models are likely to become increasingly common in the intervention evaluation literature. Similarly, we can consider any causal pathways or models which are described in the wider literature to explain similar findings, and whether these are likely to be applicable in places A and B. In addition, we can list the environmental factors from Table 1 which are pertinent to place A and place B and use these lists to develop our own hypotheses about likely casual pathways through which environmental factors influence physical activity in each place and the likely differences in these pathways. In the case of environment-based and information, education and communication-based interventions, an additional model of the processes through which the interventions are thought to operate can be developed. This can be used to make judgements about how environmental factors might interact with these processes differently in place A and place B. In addition it may be possible to carry out primary qualitative studies in place A and place B to support people with expert knowledge of places A and B (especially residents), or with expert knowledge of the processes involved in the interventions to articulate notional causal pathways.

*3. The ways in which different environmental factors influence each other in place A and place B and the differences between these*.

It is widely acknowledged that environmental factors will interact with each other in order to exert an effect on physical activity [48]. However, despite the numerous observational studies reporting associations between various combinations of environmental factors and physical activity, the ways in which environmental factors influence each other (independently of any effect on physical activity) have rarely been theorised, investigated or reported. We have proposed, giving examples, that these interactions and influences may differ between places. If this is the case then associations between environmental factors and physical activity reported in place A may not be generalisable to place B. Assessing the ways in which environmental factors influence each other in place A and place B might again involve making lists of pertinent environmental factors in each place and consulting local experts to propose which factors will influence each other in what way and in which combinations. In this case, interactions between environmental factors independent of physical activity are important, meaning that the involvement of experts from diverse disciplines such as sociology, geography, town planning and transport is likely to be useful. Similarly the application of concepts from these disciplines may illuminate the ways in which environmental factors exert influence on each other independently of physical activity. For example, the sociological 'broken windows theory' [49] describes the ways in which the aesthetic quality of the environment, crime levels, and perceived safety mutually influence each other.

## Implications of the generalisability framework for researchers, policy makers and practitioners

The framework laid out above implies that, to support judgements of generalisability, the design and conduct of research should generate high quality information on the:

- distributions of environmental factors in the research setting (place A);

- causal pathways through which information, education and communication interventions or environmental factors are thought to exert an effect on physical activity;

- ways in which different environmental factors influence each other in the settings where the research is carried out.

### Conducting research on physical activity

We have identified a need for information about the configuration of environmental factors in place A. Therefore, it is therefore necessary to make the collection of this information an integral part of the research process. This is likely to require that researchers, and those who are providing research grants, agree to allocate sufficient resources to activities that will produce this information.

We have identified a need for conceptual models that describe the causal pathways through which environmental factors and information, education and communication interventions exert an effect on physical activity. In order to achieve this, detailed case studies of places may be required in order to build comprehensive pictures of the ways in which environmental factors influence each other and interact to influence physical activity. In addition, it has been suggested that alongside quantitative research, qualitative research involving consultations with residents and local experts may best illuminate these pathways [47].

### Cross-disciplinary evidence gathering

While little is known about the ways in which environmental factors interact with each other to influence physical activity, interactions between environmental factors may have been studied extensively by other research disciplines, therefore collaboration is likely to be very valuable. For example, sociology and/or transport research may be able to inform us about the ways in which perceived safety interacts with public transport use. Or sociologists and/or environmental researchers may be able to tell us how crime rates interact with the aesthetic quality of an environment. Where information about these interactions is not available, primary research on interactions between environmental factors is likely to be desirable.

### Designing new interventions and research into their effectiveness

We have described the ways in which environmental factors may influence the causal pathways through which interventions exert an effect on physical activity, however evaluations of information, education and communication-based physical activity interventions have seldom acknowledged a role played by environmental factors. Future studies may benefit from investigating how environment factors interact with interventions to influence physical activity. This may involve the systematic consideration of which environmental factors are relevant, as we describe in the above framework. Once pertinent environmental factors have been identified and the causal pathways through which they operate conceptualised, these theories can be tested in the design and implementation of interventions and evaluations of interventions. There are a few very recent examples of how this may be achieved, for example the study by Michael and Carlson [28], which is discussed earlier under Figure 2.

### Reporting research findings

In the reporting of research findings, information about environmental factors in the research setting needs to be summarised in sufficient detail to allow a third party to judge whether the findings are generalisable between place A and place B. Research reports seldom include information about the configuration of environmental factors, the way they feed into the causal pathways through which interventions are thought to work, or the ways in which they interact. There is a clear need to improve the reporting of such information in order to allow judgement regarding the generalisability of evidence. It may be necessary for publishers to provide alternative places to make this information available (e.g. online or as appendices).

### Evidence synthesis

Systematic reviews present ideal opportunities to assess the generalisability of research. With regard to interventions, systematic reviews most often attempt to ask the question; 'does the intervention work?' and pay close attention to the internal validity of studies. However, it is becoming increasingly accepted that reviews are more useful when they also attempt to explain why interventions sometimes work and sometimes do not, the circumstances under which interventions work and the role played by environmental factors. Realist synthesis, for example integrates evidence from a number of studies to test and refine programme theory. The central principle to a realist approach to evaluation and synthesis is that the underlying assumptions about how an intervention works are made explicit and evidence is gathered systematically to test these assumptions [50]. For environment-based and information, education and communication-based interventions, an approach to synthesis that explicitly attempts to specify the role of environmental factors and incorporate theories about their role into the synthesis of evidence of the effectiveness of these interventions is likely to be extremely useful in assessing the extent to which interventions can be successfully transferred from place to place.

### Summary

In this paper we have developed a causal model and framework for assessing the generalisability of public health research evidence. We have suggested that the extent to which the main types of research evidence on physical activity can be generalised relies heavily on three principle characteristics of environmental factors in the place in which the evidence was generated and the place to which the evidence is to be generalised. Below, we have summarised recommendations to help facilitate the systematic collection and consideration of information about environmental factors in public health research for researchers, journal editors and research funding bodies:

Authors of research reports make available, either in published reports or elsewhere:

• Information about the distributions of EFs in the research setting.

• Information about the causal pathways through which IEC-based interventions or environmental factors are thought to exert their effect on PA (where appropriate).

• Information about the ways in which different environmental factors influence each other in the settings where the research is carried out.

• Information about any other factors that may act as barriers to or facilitate the process of generalising the evidence to another location.

Editors of publications presenting research reports:

• Require that the information about EFs in the research setting described above is included in research reports that are published.

• To provide adequate space (either within a report, as appendices or online) for this information to be presented.

For bodies providing research grants:

• To specify in invitations to tender that the collection of this information about EFs will be required.

• To provide funds, where appropriate, for this information to be collected.

For those designing interventions:

• To make explicit any assumptions regarding the role of EFs in the causal pathways through which the intervention is intended to exert its effect.

Our analysis and framework, although developed for physical activity, may potentially be adapted and used to consider other evidence and interventions which are likely to be sensitive to influence by elements of the structural, physical, social and/or cultural environment. These might include the epidemiology of obesity and weight management programmes. Such adaptation would require the systematic consideration of relevant environmental factors for each outcome and setting of interest, the causal pathways involved, and the typology of interventions used.

## Abbreviations

EF: Environmental factor associated with physical activity; CP: Notional or real causal pathway through which physical activity may be influenced; PA: Physical Activity.

## Competing interests

The authors declare that they have no competing interests.

## Authors' contributions

PW conceived the paper, its content and drafted the original manuscript. GP carried out significant editing and re-drafting to the final manuscript. AH provided edits to the whole paper and drafting of the discussion section. MP contributed considerably towards the conceptual content of the paper in its early stages and also provided comments on later drafts. AR contributed to the conceptual design of the paper, assisted in the development of the framework included, and carried out significant re-drafting of the manuscript. All authors read and approved the final manuscript.
